# Enzootic Sparganosis in Guangdong, People’s Republic of China

**DOI:** 10.3201/eid1508.090099

**Published:** 2009-08

**Authors:** Ming-Wei Li, Hong-Ying Lin, Wei-Tian Xie, Ming-Jian Gao, Zhi-Wei Huang, Jun-Ping Wu, Chun Li, Rui-Qing Lin, Xing-Quan Zhu

**Affiliations:** Guangdong Ocean University, Zhanjiang, People’s Republic of China (M.-W. Li, H.-Y. Lin, W.-T. Xie, M.-J. Gao, Z.-W. Huang, J.-P. Wu); South China Agricultural University, Guangzhou, People’s Republic of China (C. Li, R.-Q. Lin, X.-Q. Zhu).

**Keywords:** Sparganosis, spargana, Spirometra, frog, parasites, tapeworms, Guangdong, China, letter

**To the Editor:** Sparganosis is a worldwide parasitic zoonosis caused by infection with spargana, the plerocercoid larvae of various diphyllobothroid tapeworms belonging to the genus *Spirometra* (*1*–*3*). Sparganosis poses a serious threat to human health; the spargana invade mainly the brain, eye, abdominal cavity, spinal cord, and subcutaneous tissues; can damage local tissues; and can cause blindness, paralysis, and even death (*4*,*5*).

In the People’s Republic of China, sparganosis has emerged as an important foodborne parasitic disease, with ≈1,000 human cases reported in 22 provinces during 1927–2007. Guangdong Province has the most cases (*6*). Persons in Guangdong Province eat frog meat and place frog poultices made from raw frog meat on open wounds and lesions, which facilitates human infection with spargana. To assess the risk for human infection with sparganosis in this province and to strengthen public food safety awareness, we conducted a comprehensive investigation of spargana infection in frogs, the second intermediate host of *Spirometra*.

By necropsy we examined for spargana 544 frogs (446 *Rana nigromaculata* and 98 *R. tigrina*) from Yunfu, Maoming, and Zhanjiang in western Guangdong Province during October 2007–October 2008 (*7*). Of these 544 frogs, 455 were wild, and 89 were aquacultured. Spargana were found in 27.3% (124/455) of examined wild frogs; of these, 30.0% (107/357) were *R. nigromaculata*, significantly more (p<0.05) than the 17.3% (17/98) that were *R. tigrina*. This finding suggests that *R. nigromaculata* is the main intermediate host of *Spirometra* in western Guangdong Province.

We found 719 spargana in infected wild frogs. The number of worms per frog ranged from 1 to 41, with an average of 5.8 worms per infected frog. No spargana were found in 89 aquacultured *R. nigromaculata* frogs.

The examined wild frogs looked normal and healthy and had no obvious symptoms. During necropsy, we detected local edema, muscle bleeding, and fragile tissues in the tissues invaded by spargana. We also found cysts in some tissues that contained 1 or a few worms. Spargana dissected from host tissue were flat, white worms, which continuously crept in the normal saline. These worms ranged from 2 mm to 115 mm long and from 1 mm to 2 mm wide.

Frogs are the second intermediate hosts of *Spirometra* spp.; pigs, mice, and humans become infected as paratenic hosts by ingesting *Spirometra* larvae in cyclops or frogs (*8*,*9*). Because persons in Guangdong Province enjoy eating frog meat, particularly from wild frogs, many frogs have been sold in the market, including a substantial number of wild frogs. The results of our survey show that infection of wild frogs with spargana reached 27.3% in western Guangdong Province; hence, consumption of wild frogs (and use as poultices) poses a high risk for sparganum infection. Therefore, public health officials, epidemiologists, medical practitioners, parasitologists, veterinarians, and the general public should be aware of such risks and should implement strategies to reduce or eliminate them.
